# Strong Crystallographic Influence on Spin Hall Mechanism in PLD-Grown IrO_2_ Thin Films

**DOI:** 10.3390/nano11061478

**Published:** 2021-06-02

**Authors:** Pilar Jiménez-Cavero, Irene Lucas, Jorge Ara-Arteaga, M. Ricardo Ibarra, Pedro A. Algarabel, Luis Morellón

**Affiliations:** 1 Instituto de Nanociencia y Materiales de Aragón, Universidad de Zaragoza-CSIC, 50018 Zaragoza, Spain; ibarra@unizar.es (M.R.I.); algarabe@unizar.es (P.A.A.); morellon@unizar.es (L.M.); 2 Departamento de Física de la Materia Condensada, Universidad de Zaragoza, 50009 Zaragoza, Spain; jorgeara07arteaga@gmail.com; 3 Laboratorio de Microscopías Avanzadas, Universidad de Zaragoza, 50018 Zaragoza, Spain

**Keywords:** spin Hall effect, spin Seebeck effect, spin-to-charge conversion, iridium oxide

## Abstract

Spin-to-charge conversion is a central process in the emerging field of spintronics. One of its main applications is the electrical detection of spin currents, and for this, the inverse spin Hall effect (ISHE) has become one of the preferred methods. We studied the thickness dependence of the ISHE in iridium oxide (IrO${}_{2}$) thin films, producing spin currents by means of the spin Seebeck effect in γ−Fe${}_{2}$O${}_{3}$/IrO${}_{2}$ bilayers prepared by pulsed laser deposition (PLD). The observed ISHE charge current density, which features a maximum as a consequence of the spin diffusion length scale, follows the typical behaviour of spin-Hall-related phenomena. By fitting to the theory developed by Castel et al., we find that the spin Hall angle $\theta_{\mathrm{SH}}$ scales proportionally to the thin film resistivity, $\theta_{\mathrm{SH}}\propto \rho_{c}$, and obtains a value for the spin diffusion length $\lambda_{\mathrm{IrO}}{}_{2}$ of $\lambda_{\mathrm{IrO}}{}_{2}=3.3\left(7\right)$ nm. In addition, we observe a negative $\theta_{\mathrm{SH}}$ for every studied thickness and temperature, unlike previously reported works, which brings the possibility of tuning the desired functionality of high-resistance spin-Hall-based devices. We attribute this behaviour to the textured growth of the sample in the context of a highly anisotropic value of the spin Hall conductivity in this material.

## 1. Introduction

The spin Hall effect (SHE) refers to the creation of a spin current transverse to a charge current in a nanometric metallic material [1,2,3,4,5]. It emerges in materials with high spin-orbit coupling (SOC), which endows electrons with a spin-dependent component of velocity perpendicular to the charge current. The specification for nanodimensions is due to the magnitude of the decay lengths of spin currents [6]. The reciprocal of SHE is known as the inverse spin Hall effect (ISHE). It appears as a conventional charge current induced by a transverse spin current in metallic nanostructured materials with high SOC [5,7,8]. This charge current leads to charge accumulation at the edges of the device that can be easily detected as an electrical voltage. The spin-to-charge conversion by the ISHE is thus one of the preferred methods for spin current detection. An important parameter in this regard is the spin Hall angle $\theta_{\mathrm{SH}}$, which determines the efficiency of the spin-to-charge conversion:

(1) $$\begin{array}{c} \mathrm{SHE}:\;\;\;\;\;\;\;\;\;J_{s}=\theta_{\mathrm{SH}}\frac{\hbar }{2e}J_{c}\times s\\ \mathrm{ISHE}:\;\;\;\;\;\;\;\;\;J_{c}=\theta_{\mathrm{SH}}\frac{2e}{\hbar }J_{s}\times s, \end{array}$$

where $J_{s}$ and $J_{c}$ denote the spin and charge current densities, respectively, and s is the spin polarization. The spin Hall conductivity $\sigma_{\mathrm{SH}}$ in a metal is here defined as $\sigma_{\mathrm{SH}}=\sigma_{xy}^{↑}-\sigma_{xy}^{↓}$, where $\sigma_{xy}$ is the xy component of the conductivity tensor for up-spin polarized and down-spin polarized carriers. This transverse conductivity is related to its resistivity counterpart $\rho_{xy}$ by

(2) $$\sigma_{xy}=-\frac{\rho_{xy}}{\rho_{xx}^{2}+\rho_{xy}^{2}}\approx -\frac{\rho_{xy}}{\rho_{xx}^{2}},$$

where $\rho_{xx}$ is the longitudinal resistivity and we have taken the limit $\rho_{xy}\ll \rho_{xx}$ [9,10]. Renaming the longitudinal electrical conductivity and resistivity as $\sigma_{\mathrm{xx}}\equiv \sigma_{c}$ and $\rho_{\mathrm{xx}}\equiv \rho_{c}$, the spin Hall angle can be expressed as

(3) $$\theta_{\mathrm{SH}}=\frac{\sigma_{\mathrm{SH}}}{\sigma_{c}}\approx -\frac{\rho_{\mathrm{SH}}}{\rho_{c}},$$

where $\rho_{\mathrm{SH}}$ is the spin Hall resistivity.

The SHE and ISHE arise from extrinsic and intrinsic microscopic mechanisms [10,11]. The extrinsic SHE/ISHE results from spin-asymmetric scattering at impurities, boundaries or defects (non-periodic or disorder potential) [1,2,3,12]. Skew scattering [13] and side jump [14] mechanisms are recognized as sources of spin-dependent displacement of electrons during scattering events: skew scattering is the dependence of the scattering angle sign on the electron spin, and side jump refers to a transverse shift of the electron trajectory that is dependent on the spin. The intrinsic contribution to the SHE/ISHE occurs between scattering events and arises from the band structure of the perfect crystal (periodic or lattice potential) [15,16].

Usually, heavy transition metals, such as Au or Pt, are employed for spin-to-current conversion by the SHE or ISHE. However, these noble metals show extremely low electrical resistivity $\rho_{c}$, and whereas this fact represents an advantage when they are used for spin current injection (by the SHE), it degrades their performance in spin current detection (by the ISHE) since the generated voltage $\Delta V_{\mathrm{ISHE}}$ is proportional to $\rho_{c}$ [8]:

(4) $$\Delta V_{\mathrm{ISHE}}\propto \theta_{\mathrm{SH}}\rho_{c}J_{s}\approx \rho_{\mathrm{SH}}J_{s}.$$

In the search for materials with good performance as spin current detectors via the ISHE, 5d transition metal oxides (TMOs) have attracted the interest of the community because of their strong SOC [17] and moderate electrical conductivities [18,19]. In particular, the family of iridates shows intriguing phenomena such as metal-insulator transitions [20,21], exotic magnetic ground states [22,23] or novel topological phenomena [21,24,25]. The parental compound IrO${}_{2}$, a metallic material showing no magnetic order, has been targeted as a highly valid spin current detector. Fujiwara and coworkers studied the performance of polycrystalline and amorphous samples of IrO${}_{2}$ in spin absorption experiments carried out with non-local spin-valve structures [19], finding a value for $\rho_{\mathrm{SH}}$ one order of magnitude larger than those of noble metals. In contrast, Qiu et al. studied the performance of IrO${}_{2}$ in a longitudinal spin Seebeck effect (LSSE) device and found it to be significantly lower than that of Pt [26]. In an LSSE experiment, a spin current was excited in a magnetically ordered material (FM) as a consequence of the application of a thermal gradient [27,28,29]. The current consensus is that this spin Seebeck-spin current is transported by the collective excitations of local moments (magnons) [28,30], and it is therefore better studied in insulators where there is no contribution of free carriers to the output. The thermal spin current is injected into an adjacent non-magnetic (NM) material (interface normal to the spin current, which in turn is parallel to the applied thermal gradient), where it is converted into a transverse charge current by means of the ISHE. Qiu et al. [26] attributed the small signal of the ISHE found in IrO${}_{2}$ in their experiment to a low spin mixing conductance, a parameter that quantifies the efficiency of the transmission of spin across the interface between two layers.

The electronic band structure of IrO${}_{2}$ has been theoretically [31,32,33,34,35,36,36] and experimentally [17,33,36,37,38,39,40,41] addressed as early as 1977 [42]; nevertheless, it was the experimental evidence of very efficient spin-to-charge conversion [19] that renewed interest in this fascinating material and triggered many works dedicated to explaining the large SHE/ISHE. However, the precise role of the different SHE and ISHE mechanisms in IrO${}_{2}$ has not yet been clearly elucidated.

In this paper, we performed a systematic study of the ISHE detection of the SSE in γ–Fe${}_{2}$O${}_{3}$/IrO${}_{2}$ bilayer structures for different thicknesses of the IrO${}_{2}$ layer, ranging from 2 to 22 nm. We obtained the value of the spin diffusion length of IrO${}_{2}$ and found that the spin Hall angle is proportional to the longitudinal charge resistivity, $\theta_{\mathrm{SH}}\propto \rho_{c}$; equivalently, the value of $\sigma_{\mathrm{SH}}$ is constant and independent of the longitudinal charge conductivity $\sigma_{c}$, and $\rho_{\mathrm{SH}}$ scales as $\rho_{\mathrm{SH}}\propto \rho_{c}^{2}$ (see Equations (2) and (3)).

## 2. Materials and Methods

γ−Fe${}_{2}$O${}_{3}$/IrO${}_{2}$ bilayers were grown using the pulsed laser deposition technique on Al${}_{2}$O${}_{3}$(0001) substrates. Both layers were consecutively in situ deposited in the same vacuum chamber without exposure to room atmosphere in between. Each sample was prepared according to the following procedure: first, PLD was used to deposit a layer from a 99.99% pure Fe${}_{3}$O${}_{4}$ (magnetite) target under vacuum conditions. The repetition rate of the KrF excimer 248 nm wavelength laser was set to 10 Hz with a 3.7 J/cm${}^{2}$ fluence. The base pressure in the deposition chamber was ∼10${}^{-8}$ Torr. Second, the Fe${}_{3}$O${}_{4}$ layer was in situ annealed in an oxygen atmosphere ($P_{O_{2}}=50$ mTorr) at 325 ℃ to transform it into the γ−Fe${}_{2}$O${}_{3}$ phase (maghemite), a ferrimagnetic insulator [43]. Finally, the IrO${}_{2}$ detection layer was PLD grown at the same temperature and oxygen pressure by striking a 99.9% pure IrO${}_{2}$ target with a fluence of 1.6 J/cm${}^{2}$ at a 3 Hz repetition rate. The thickness of maghemite was kept at 50 nm throughout every sample, whereas the thickness of IrO${}_{2}$, $t_{\mathrm{IrO}}{}_{2}$, was varied from 2 to 22 nm.

X-ray characterization of the samples was performed in a high-resolution Bruker D8 Advance diffractometer Prior to LSSE experiments, the longitudinal resistivities $\rho_{c}$ of the different IrO${}_{2}$ layers were determined using an in-line four probe geometry to ensure the metallic behaviour of IrO${}_{2}$ thin films, measuring the V(I) characteristic curves between ±20μA.

## 3. Results and Discussion

**Figure 1:**
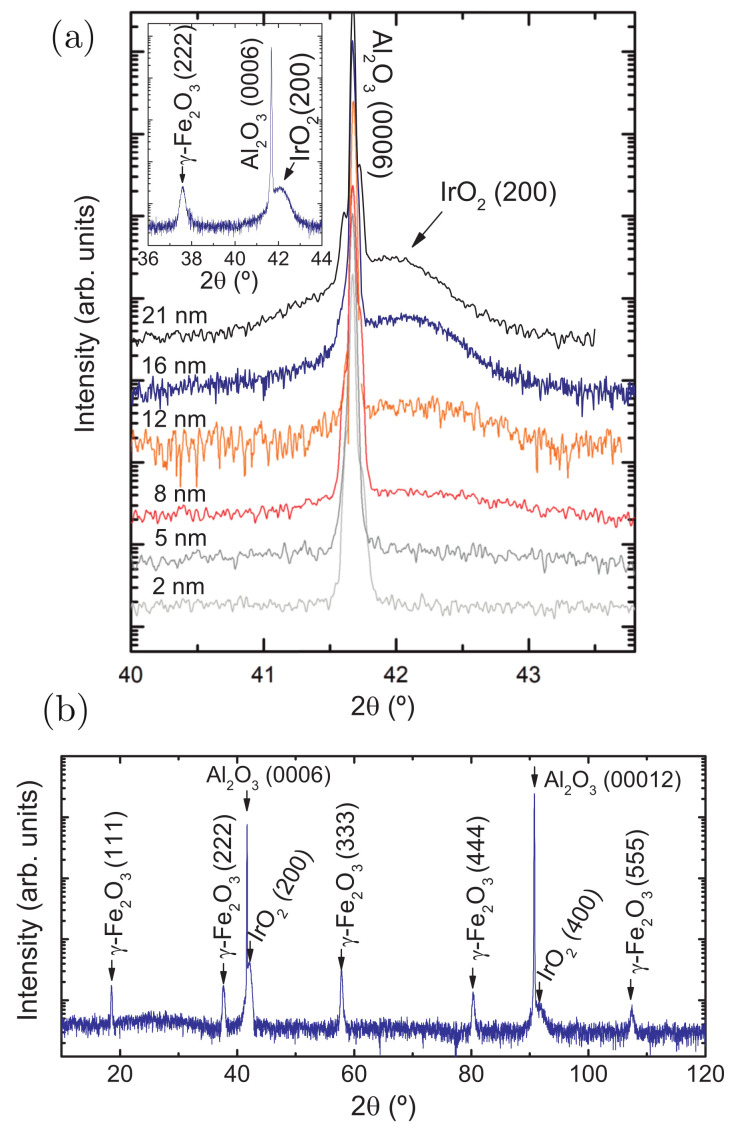
(**a**) Symmetric 2θ/ω diffraction patterns around the (0006) Al${}_{2}$O${}_{3}$ Bragg peak. Inset: longer-range measurement for the sample with a 16 nm-thick IrO${}_{2}$ layer, including the (222) diffraction peak of γ−Fe${}_{2}$O${}_{3}$; (**b**) Wide-range symmetric 2θ/ω XRD scan for the sample with a 16 nm-thick IrO${}_{2}$ layer.

The 2θ/ω scans around the (0006) substrate Bragg peak are shown in Figure 1a. The (200) IrO${}_{2}$ peak is visible and increases in intensity as the layer thickness is increased. In Figure 1b, a wide-range 2θ/ω scan for one of the samples is also provided: only the {100} reflections of IrO${}_{2}$ are present, indicating that this sample is strongly textured in this direction. Scherrer’s empirical formula [44] was applied to the diffraction peak of the two thickest samples ($t_{\mathrm{Iro}}{}_{2}$= 22 and 16 nm) in order to determine the crystalline domain size of IrO${}_{2}$ in the [100] direction, yielding ≈13 nm and ≈11 nm. The domain size is smaller than the respective thicknesses of the IrO${}_{2}$ layer, and therefore, the single-crystalline orientation must be discarded. However, the obtained crystal coherence lengths are close to the thicknesses, indicating a texture preference for the [100] direction. Regarding γ−Fe${}_{2}$O${}_{3}$, it grows on top of Al${}_{2}$O${}_{3}$ in the [111] orientation; the inset in Figure 1 displays, for one of the samples, a more extended range including the (222) Bragg peak of maghemite as an illustrative example. This was also confirmed by the longer-range pattern presented in Figure 1b, where only the {111} reflections of γ−Fe${}_{2}$O${}_{3}$ are visible.

**Figure 2:**
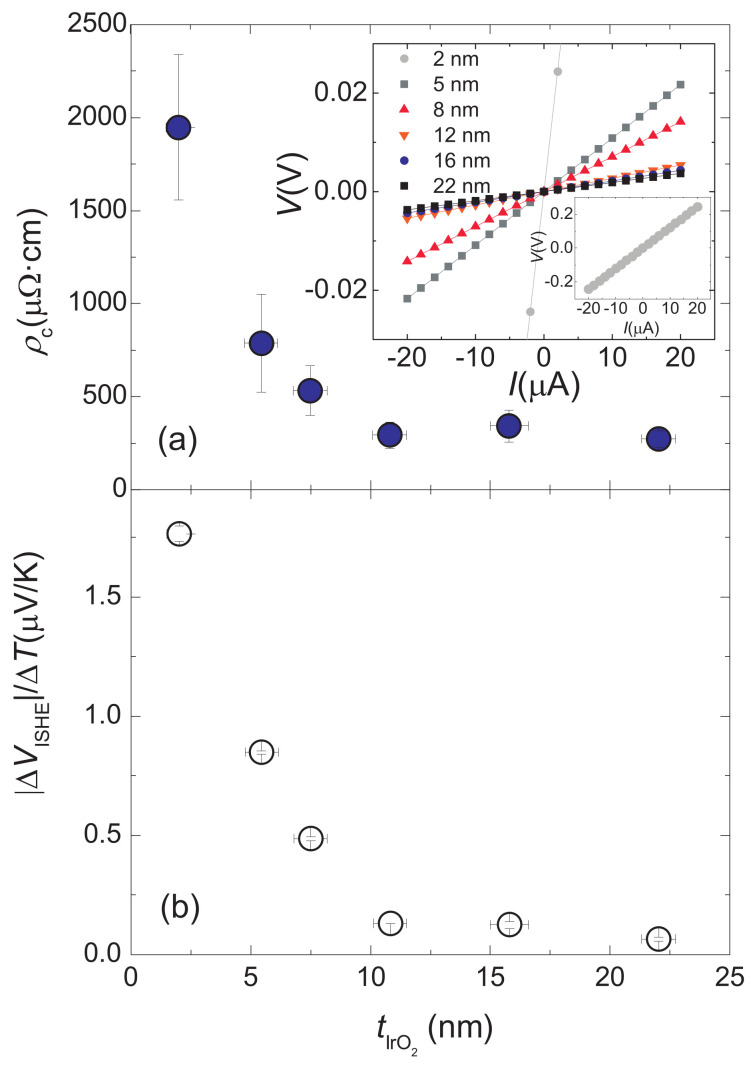
(**a**) Longitudinal resistivities of samples. The V(I) characteristic curves measured to determine the electrical resistances of the IrO${}_{2}$ thin films are displayed in the inset. The V(I) characteristic curve of the thinner sample is zoomed out; (**b**) Voltage detected via the ISHE from LSSE experiments.

The measured I(V) characteristic curves displayed ohmic behaviour for all samples (see the inset of Figure 2a), which proves the metallic nature of IrO${}_{2}$. As shown in Figure 2a, the resistivity increases with decreasing thickness, a fact that may suggest a variable density of defects as the growth progresses. However, this behaviour is also consistent with recent theoretical works that predict changes in the metallic properties of IrO${}_{2}$ through thickness variation [45] and epitaxial strain along the *c* axis [33].

**Figure 3:**
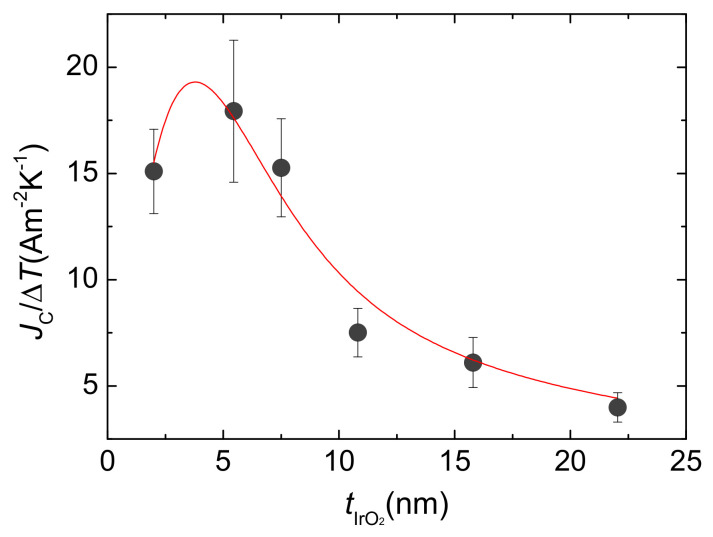
Symbols: ISHE current density normalized by the thermal drop through the sample for every IrO${}_{2}$ thickness. Line: fit to Equation (6).

For every γ−Fe${}_{2}$O${}_{3}$/IrO${}_{2}$ bilayer, we measured the output voltages $\Delta V_{\mathrm{ISHE}}$ for different thermal drops ΔT applied to thermally excite spin currents by means of the LSSE. Following a widespread practice, these quantities have been normalized by the total thermal difference ΔT using the slopes of the linear fits of $\Delta V_{\mathrm{ISHE}}$ as a function of different ΔT. The data of the output voltages $\Delta V_{\mathrm{ISHE}}$ measured per applied Kelvin for every thickness of IrO${}_{2}$ are depicted in Figure 2b. The common behaviour of $\Delta V_{\mathrm{ISHE}}/\Delta T$ and $\rho_{c}$ indicates that $\rho_{c}\left(t_{\mathrm{IrO}}{}_{2}\right)$ strongly dominates the $\Delta V_{\mathrm{ISHE}}/\Delta T$ dependence on $t_{\mathrm{IrO}}{}_{2}$. However, the contribution to this dependence that interests us is that of the spin-to-charge conversion process, as expressed in Equation (1). Therefore, we calculate $J_{c}$ as $J_{c}/\Delta T=\left(\Delta V_{\mathrm{ISHE}}/\Delta T\right)/\left(d_{y}\cdot \rho_{c}\right)$, where $d_{y}$ represents the distance between the electrical contacts used to measure $\Delta V_{\mathrm{ISHE}}$. In this way, we eliminate the influence of $\rho_{c}$ on the thickness dependence of Equation (4) and focus on that of $\theta_{\mathrm{SH}}J_{s}$. The obtained values are plotted in Figure 3. We note that $J_{c}/\Delta T$ indeed features a peak at low values of $t_{\mathrm{IrO}}{}_{2}$ followed by a monotonic decrease for higher values. This behaviour constitutes the fingerprint of a typical diffusion mechanism with a characteristic length, $\lambda_{\mathrm{IrO}}{}_{2}$, comparable to the layer thickness [6]. This curve is similar to that reported for other ISHE media, such as prototypical Pt [46,47].

To extract quantitative information about $\lambda_{\mathrm{IrO}}{}_{2}$, we make use of the model developed by Castel et al., which relates the thickness of an NM layer to the detected transverse ISHE voltage caused by a spin current [46]. According to this model, the NM thickness (*t*) dependence of $\Delta V_{\mathrm{ISHE}}$ can be expressed as

(5) $$\Delta V_{\mathrm{ISHE}}\propto \frac{\theta_{\mathrm{SH}}}{t}\cdot \frac{g_{↑↓}}{g_{↑↓}+\frac{1}{\lambda \rho_{c}}\cdot \frac{1-e^{-2t/\lambda }}{1+e^{-2t/\lambda }}}\cdot \frac{{\left(1-e^{t/\lambda }\right)}^{2}}{1+e^{2t/\lambda }},$$

where $\theta_{\mathrm{SH}}$ represents the spin-Hall angle, $g_{↑↓}$ is the spin mixing conductance of the FM/NM interface, λ denotes the spin diffusion length of the NM layer and $\rho_{c}$ is its electrical resistivity. Equivalently:

(6) $$J_{c}\propto \frac{\theta_{\mathrm{SH}}}{t\rho_{c}}\cdot \frac{g_{↑↓}}{g_{↑↓}+\frac{1}{\lambda \rho_{c}}\cdot \frac{1-e^{-2t/\lambda }}{1+e^{-2t/\lambda }}}\cdot \frac{{\left(1-e^{t/\lambda }\right)}^{2}}{1+e^{2t/\lambda }}.$$

In their work [46], based on spin-pumping experiments with YIG/Pt bilayers, Castel and coworkers supposed that the ISHE originated from extrinsic mechanisms due to skew scattering and therefore $\sigma_{\mathrm{SH}}\propto \sigma_{c}$, yielding a constant $\theta_{\mathrm{SH}}$ [9,10,11]. However, such an assumption does not explain the observed experimental results. Equation (5) will only adequately describe the experimental data when taking $\theta_{\mathrm{SH}}\propto \rho_{c}=\sigma_{c}^{-1}$ (i.e., $\sigma_{\mathrm{SH}}$ is independent of $\sigma_{c}$ and $\rho_{\mathrm{SH}}\propto \rho_{c}^{2}$). Admitting this scenario, a fit of Equation (6) to the current density data converges, providing a value for the spin diffusion length of IrO${}_{2}$ of $\lambda_{\mathrm{IrO}}{}_{2}=3.3\left(7\right)$ nm. This result rules out a dominant role of skew scattering in the ISHE process, as this mechanism is characterized by a constant $\theta_{\mathrm{SH}}$ [9,10,11].

**Figure 4:**
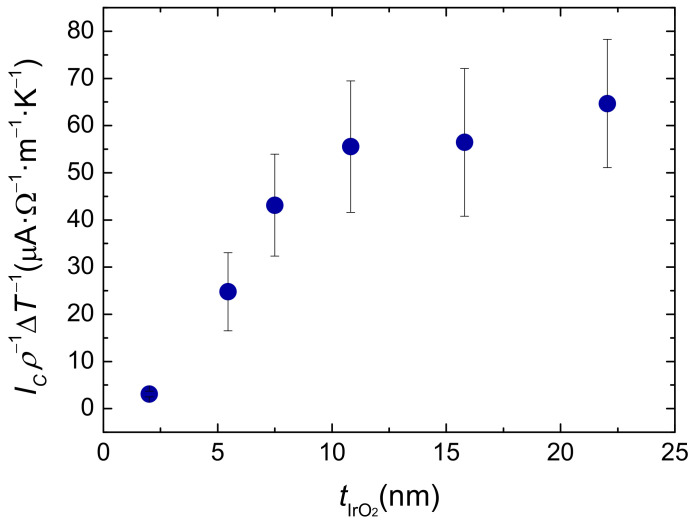
Evolution of $I_{c}/\left(\rho_{c}\Delta T\right)$ with the thickness of IrO${}_{2}$.

The behaviour of the absolute ISHE charge current $I_{c}$ above the spin diffusion length also provides information about the mechanism responsible for the ISHE. If skew scattering is the main term, assuming that the injected spin current $I_{s}$ is the same for all samples (since $t_{\mathrm{FM}}$ is maintained constant), then $\theta_{\mathrm{SH}}$ would be independent of $\rho_{c}$ and thus, of $t_{\mathrm{IrO}}{}_{2}$, which would make $I_{c}$ saturate to a constant value for $t_{\mathrm{IrO}}{}_{2}>\lambda_{\mathrm{IrO}}{}_{2}$. In contrast, in the case that the spin Hall angle scales as $\theta_{\mathrm{SH}}\propto \rho_{c}$, $I_{c}$ needs to be normalized by $\rho_{c}$ to observe a saturation level, as the efficiency of the spin-to-charge conversion (represented by $\theta_{\mathrm{SH}}$) increases with increasing $\rho_{c}$. As shown in Figure 4, the experimental data follow this latter trend, supporting the $\theta_{\mathrm{SH}}\propto \rho_{c}$ scaling and thus excluding skew scattering as the main ISHE mechanism in the experiment.

Therefore, these results entail that either the intrinsic mechanism or the extrinsic side jump must govern the ISHE in IrO${}_{2}$ thin films with [100] preferential texture because both of them share the same $\theta_{\mathrm{SH}}$ dependence on resistivity [9,10,11,14].

However, the separation of intrinsic and side jump contributions has been a long-standing problem, also controversial from a theoretical point of view. Some authors [48] predicted that a side jump is always negligible compared to skew scattering (by a factor of $1/Z^{2}$); in contrast, others concluded that both contributions can be comparable [49,50]. In either case, the intrinsic contribution is the most likely candidate to play the dominant role in our experiment, as we discuss in the following.

First, if we accept the result in reference [48]—side jump is generally negligible relative to skew scattering—considering that we have ruled out a predominant role of skew scattering, we can conclude that the intrinsic SOC governs the ISHE. Alternatively, we examine the situation that aligns with the results in [49,50,51]—that the side jump is not always negligible. In this regard, we recall that the side jump contribution to $\theta_{\mathrm{SH}}$ is proportional to the impurity concentration [11,49]; this is why, although it may dominate the overall effect at sufficiently high impurity concentrations, its contribution to the spin Hall conductivity is usually smaller than those of skew scattering or the intrinsic mechanism [11]. As a consequence, the side jump is usually manifested either in doped systems or alloys (not our case) or at low temperatures, where it gains importance and may become comparable to skew scattering even at low impurity concentrations [51,52,53]. Nevertheless, our experiments were performed at room temperature. Additionally, Fert and Levy showed that for impurities at the beginning and end of the 5d series (Lu, Hf, Ir, Pt), the side jump contribution at impurity concentrations of ≈2% is much smaller than the skew scattering contribution [49]. In view of all of the above, it seems reasonable to accept that the ISHE in thin films of IrO${}_{2}$ is most likely driven by the intrinsic SOC.

**Figure 5:**
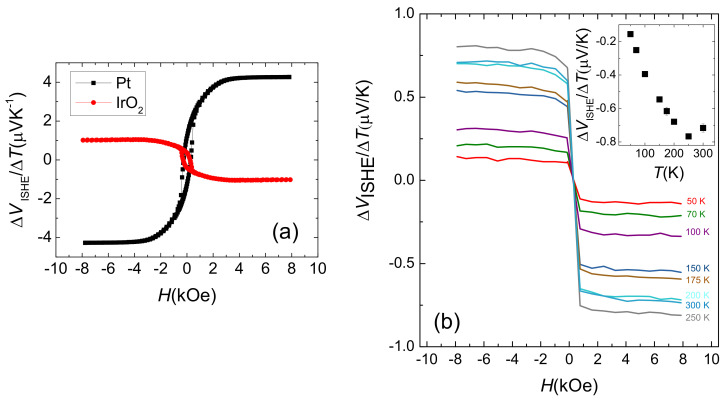
(**a**) Comparison between the $\Delta V_{\mathrm{ISHE}}$ measured as a function of the magnetic field for γ–Fe${}_{2}$O${}_{3}$/Pt bilayer and a γ–Fe${}_{2}$O${}_{3}$/IrO${}_{2}$ bilayer; (**b**) Evolution with temperature of the measured $\Delta V_{\mathrm{ISHE}}$ output excited by the LSSE in the sample with $t_{\mathrm{IrO}}{}_{2}=5.5$ nm.

Another relevant observation concerns the sign of $\Delta V_{\mathrm{ISHE}}$: it is negative, entailing $\theta_{\mathrm{SH}}<0$. To unambiguously define the sign of the ISHE in our setup, a γ–Fe${}_{2}$O${}_{3}$/Pt bilayer was used. In Figure 5a, we compare two measurements performed in γ–Fe${}_{2}$O${}_{3}$/Pt and in γ–Fe${}_{2}$O${}_{3}$/IrO${}_{2}$, to evidence that the corresponding transverse voltages display opposite signs. In sight of this, the negative sign of $\Delta V_{\mathrm{ISHE}}$ is necessarily originated by a negative value of $\theta_{\mathrm{SH}}$, as evidenced by Equation (4).

In the pioneering work with polycrystalline IrO${}_{2}$ of Fujiwara and coworkers [19], they observed a change in the sign of the ISHE signal with decreasing temperature (from positive at T>90 K to negative at T<90 K). They ascribed this to the coexistence of different SOC mechanisms with opposite signs. We also performed the LSSE experiment at different temperatures for the sample in which the largest room temperature value of $J_{c}/\Delta T$ was measured ($t_{\mathrm{IrO}}{}_{2}=5.5$ nm). The results are plotted in Figure 5b. As shown, we do not observe such a reversal in the signal with respect to the magnetic field, which defines the magnetization sign of γ−Fe${}_{2}$O${}_{3}$; rather, it is negative for every measured temperature. This suggests that the same ISHE mechanism dominates over the entire temperature range.

Regarding this, it was also recently shown that the intrinsic spin Hall conductivity $\sigma_{ij}^{k}$ in IrO${}_{2}$ is remarkably anisotropic, changing not only in magnitude, but also in sign depending on the directions of the spin current (*i* direction), spin polarization (*k* direction) and electric field (*j* direction) [35]. As a result, the sign of the induced ISHE electric field $E_{j}$ depends not only on the sign of the vector product $J_{s}^{i}\times s$, but also on the direction in which the spin-to-charge conversion process is occurring. Accordingly, the preferred direction (if any) of growth with respect to the measurement geometry might be determining for the observed sign if the intrinsic mechanism is dominant. This means that the sample preparation and crystallinity are probably crucial for the final balance in the competition between different mechanisms to dominate the ISHE in IrO${}_{2}$. Thus, the explanation for the differences observed between samples with [100] preferential texture and previously reported results on polycrystalline IrO${}_{2}$ may be found here. Further measurements performed in IrO${}_{2}$ layers with other crystal orientations are needed to confirm this interpretation. The samples studied in reference [19], polycrystalline, were prepared by reactive sputtering from a pure Ir target and then patterned using e-beam lithography. Qiu and collaborators in reference [26] mentioned that they used RF sputtering. Competition between SOC mechanisms opposite in sign but similar in magnitude could be responsible for the low ISHE signal they observed in their experiment, together with a low spin mixing conductance. Very recently, Bose et al. experimentally determined by means of spin-torque ferromagnetic resonance that the ISHE regimen for epitaxial (001) IrO${}_{2}$ films was different to that of (110)-oriented films [54]. To the best of our knowledge, there are no reported results of ISHE experiments on textured thin films of IrO${}_{2}$ or on PLD-grown films.

## 4. Conclusions

In summary, we investigated the ISHE spin-to-charge conversion in PLD-grown thin films of IrO${}_{2}$. We thermally excited spin currents by making use of the LSSE and measured the transverse ISHE voltage. First, we studied the IrO${}_{2}$ thickness dependence of the process. The analysis of the obtained data within the theoretical model allowed us to establish that the spin Hall angle scales with the longitudinal charge resistivity as $\theta_{\mathrm{SH}}\propto \rho_{c}$, which excludes a predominant role of skew scattering in the ISHE. The fitting of the theoretical model in Ref. [46] to $J_{c}\left(t_{\mathrm{IrO}}{}_{2}\right)$ yields a spin diffusion length of $\lambda_{\mathrm{IrO}}{}_{2}=3.3\left(7\right)$ nm, which is in accordance with the value previously reported by Fujiwara using a lateral spin valve methodology [19]. This spin diffusion length is very comparable to that of pure metals, such as those reported for prototypical Pt, which ranges between 1.2 and 8.0 nm (see, for example, Refs. [46,55,56,57,58,59]). Second, we observed a negative sign for the spin Hall angle throughout all our experiments, including temperature variation. This is in contrast to what was described in the other two works on the ISHE in polycrystalline or amorphous IrO${}_{2}$, grown using other techniques. We attribute this to the pre-eminence of the intrinsic ISHE in the entire temperature range, with a negative intrinsic spin Hall conductivity, as proposed by [35]. This effect might be enhanced by the textured growth of IrO${}_{2}$ thin films, in view of the anisotropic nature predicted for IrO${}_{2}$ [35]. These results are relevant for the achievement of the better control of spin-to-charge conversion in this material, which shows great potential to be exploited in the spintronics field, once a deeper understanding of how the SOC in it works is attained.

## Data Availability

Data are available from the corresponding author upon reasonable request.
